# Anti-CD20 monoclonal antibodies for idiopathic nephrotic syndrome: Advances, challenges, and future directions

**DOI:** 10.1007/s00467-025-06738-w

**Published:** 2025-05-01

**Authors:** Andrea Angeletti, Gian Marco Ghiggeri

**Affiliations:** Nephrology, Dialysis, and Transplantation, IRCCS Istituto Giannina Gaslini, Via Gaslini 6, Genoa, Italy

Anti-CD20 monoclonal antibodies (anti-CD20) are a class of therapeutic agents developed over the past three decades, characterized by specific mechanisms of action and broad spectrum of clinical indications [1]. Clinical research, along with occasional setbacks, has played a pivotal role in establishing the use of anti-CD20 antibodies for pathological conditions associated with abnormal CD20 + B-cell proliferation or indirectly linked to excessive antibody production. Anti-CD20 antibodies are now most commonly used in the treatment of lymphoproliferative blood disorders and a broad spectrum of autoimmune diseases [2, 3].

The chimeric anti-CD20 antibody rituximab was the first to be developed and still represents the most widely administered in clinical practice. Second-generation anti-CD20 antibodies, including humanized (ofatumumab) and fully human (obinutuzumab) agents, are newer additions that require further evidence to solidify their indications. The article by Zhi et al., in this issue of *Pediatric Nephrology* [4], focuses on the use of rituximab in a medium-to-large cohort of children with idiopathic nephrotic syndrome (NS), providing detailed guidance on its optimal use for managing steroid-dependent and frequently relapsing cases. Additionally, it offers a broader perspective on how clinical experience and mechanistic insights have positioned anti-CD20 antibodies as fundamental tools in the treatment of idiopathic NS.

## Rituximab: targets and effects

Rituximab is a chimeric monoclonal depleting antibody targeting the CD20 antigen expressed on B cells early during maturation [1]. In addition to the CD20 antigen, that is the major target, B cells express other antigens with comparable affinity for rituximab, i.e., CD19, CD38, CD24, and IgD; they appear after CD20 during the maturation steps and are gradually lost later during B cell development. Upon binding, B cells are killed through different mechanisms, including direct or antibody-mediated cytotoxicity, phagocytosis, and complement-mediated cytolysis [5]. The lowering effect of rituximab on CD20-B cells is delayed over time with a median of 6 months, whereas the effect on B cells at different stages of maturation, such as memory B cells, perdures for more than 1 year.

Despite B cells serving as the primary source of IgG immunoglobulins and playing a central role in orchestrating the immune response, the benefits of anti-CD20 therapies extend beyond this function. An example of non-canonical B-cell effects is the induction of regulatory T cells (Treg), a cell subset that is crucial for the control of autoreactive conventional T cells. The functions of Treg include the production of pro-inflammatory and regulatory cytokines, antigen presentation to T cells and direct stimulation of T cell activation. These diverse activities position B cells as key players not only in humoral immunity but also in the regulation of cellular immune processes.

Therefore, anti-CD20 monoclonal antibodies deplete B cells, inhibit production of cytokines, disrupt antigen presentation, and modulate T cell responses, thereby dampening multiple arms of the immune system. This may explain the efficacy of anti-CD20 therapies in diseases where circulating autoantibodies are rare, absent, or not directly implicated. In rheumatoid arthritis, for example, the inhibitory effect of rituximab on intra-articular BAFF (a member of the TNF family) is associated with significant clinical improvement of local symptoms typical of this condition [6].

In accordance with its multiple functions, rituximab was firstly approved by the US Food and Drug Administration (FDA) for the treatment of relapsing or refractory non-Hodgkin’s lymphoma and chronic lymphocytic leukemia [2]. In more recent years, rituximab was licensed for typical antibody-mediated and inflammatory diseases such as lupus erythematosus, autoimmune porpora, rheumatoid arthritis, and granulomatosis with polyangiitis, in spite of the response to rituximab monotherapy in all these conditions being more heterogeneous and less intense than in other diseases [3].

## Rituximab in idiopathic nephrotic syndrome

In 2004, a child with active NS underwent proteinuria remission after receiving rituximab to treat supervened idiopathic thrombocytopenic purpura [7]. After this initial case, other reports from patients receiving rituximab for a post-transplant lymphoproliferative disorder (PTLD) indicated a potential benefit also on post-transplant NS recurrence [8, 9]. Subsequently, there have been many case series reporting rituximab efficacy in patients with steroid dependent (SD) and frequently relapsing nephrotic syndrome (FRNS) [10–15]. Moreover, several randomized clinical trials compared rituximab to the immunosuppressive drugs, such as calcineurin inhibitors and mycophenolate mofetil, usually administered as steroid sparing agents in the treatment of steroid sensitive NS (SSNS) [16–21]. On the other hand, the efficacy of rituximab in inducing remission in steroid resistant NS (SRNS) is limited to 25–35% of cases [17].

In terms of safety, rituximab is generally well tolerated, with infusion-related reactions being the most common complication, occurring in 5–10% of cases that can be minimized with slow infusion and pre-treatment using steroids, antihistamines, paracetamol, and salbutamol. Moreover, subcutaneous administration may further reduce infusion-related reactions [22].

Serious adverse events in children with NS receiving rituximab are rare but include isolated reports of pulmonary fibrosis and fulminant myocarditis. Previous studies have reported hypogammaglobulinemia in approximately 35% of patients treated with rituximab. However, while hypogammaglobulinemia can persist for months after the last infusion, its association with an increased risk of severe infections remains unclear and warrants further investigation [23].

## Recurrence and timing of rituximab re-treatment

Prediction of response to rituximab is a central subject of clinical research, especially in conditions of difficult phenotype definition, such as multiple sclerosis. In this case, depletion of a subset of memory B cells (i.e., CD19 + /CD27 +) was associated with persisting relapse for over 12 months and represents a parameter of clinical utility [24]. The ability to predict rituximab efficacy in NS and, mostly, the proteinuria recurrence are critical challenges that could significantly refine the therapeutic approach. A crucial point is anticipating NS relapse before the appearance of proteinuria that may influence timing and dosing of rituximab infusions.

A recent retrospective study by Colucci et al. suggested that in patients with SSNS receiving rituximab, a lower risk of relapse was significantly associated with an older age (over 9.8 years) [25, 26] and with memory B cell counts. Therefore, both younger age and higher circulating memory B cells at time of infusion were independently associated with a higher risk of relapse and an earlier recovery of memory B cells following anti-CD20 treatment in children with FR/SDNS is proposed to define a correct timing of infusions.

An extensive literature considered different dosing models and robust conclusions were reached from retrospective studies suggesting that single infusions of rituximab have similar effects of multiple infusions per cycle. Chan et al. [27, 28], in a retrospective multicenter study on 511 pediatric patients with SD/FRNS, investigated the effect of rituximab prescribed at three dosing levels per course: low (375 mg/m^2^), medium (750 mg/m^2^), and high (1125–1500 mg/m^2^), with or without maintenance immunosuppression. As main results, without maintenance immunosuppression, low-dose rituximab had a shorter relapse-free period than medium and high doses, while in maintenance immunosuppression, the relapse-free survival in low-dose rituximab was similar to other doses [27]. Of interest, the same authors in a different large retrospective study reported that pediatric FR/SDNS receiving repeated courses of rituximab experience an improving clinical response [28, 29].

Overall, previous randomized prospective and retrospective experiences suggest that in pediatric FR/SDNS rituximab is effective in maintaining the disease free-remission also when administered without the maintenance of oral immunosuppressive drugs, and those memory B-cell subsets may represent a reliable marker of disease recurrence.

The study by Zhi et al. in this issue [4] provides possible relevant insights, reporting two independent predictors of disease recurrence: a reduced NK cell percentage at the initiation of rituximab and a prior diagnosis of SRNS. Moreover, the study suggests the clinical significance of monitoring CD20 + B-cell repopulation. Patients who exhibited CD20 + B-cell counts exceeding 1% of total lymphocytes following rituximab treatment received a second infusion, achieving remission of NS for a minimum of 2 years in half of cases. In contrast, sustained CD20 + B-cell levels below the 1% threshold after the initial rituximab administration were associated with prolonged remission. These findings highlighted the importance of precise immunological monitoring in optimizing treatment strategies and predicting long-term outcomes.

Circulating NK cells may play a regulatory role in CD20 + B-cell repopulation, potentially slowing the process. In their absence or in cases of reduced NK cell activity, CD20 + B-cell repopulation could occur more rapidly. Studies have shown that children with SDNS exhibit both quantitative and qualitative abnormalities in NK cells compared to healthy peers, including reduced levels of CD56dim NK cells (cytotoxic subset) and increased CD56bright NK cells (regulatory subset). Interestingly, these abnormalities are positively influenced by rituximab therapy [30].

The observation of improved response to a second rituximab infusion in patients with faster CD20 + B-cell repopulation aligns with their low NK cell levels, reinforcing the interplay between NK cells and CD20 + B cells in the recurrence of NS. One plausible mechanism is that the cytotoxic effect of rituximab on B cells is partially mediated by NK cells via antibody-dependent cellular cytotoxicity.

However, the conclusions by Zhi et al. should be mitigated by the retrospective nature of study, and above all, by the cohort type: all patients were in steroids treatment at the rituximab infusion and 50/89 (56%) were also receiving maintenance oral immunosuppressive drugs (MMF, CNI, or both). Moreover, the concomitant immunosuppressive treatments were withdrawn in a significantly different time, ranging from 3 to 6 months after rituximab treatment. Therefore, the reduced NK cell percentage that correlated with disease recurrence may, at least in part, reflect the different immunosuppressive regimen among the cohort; indeed, CNI and MMF directly influence NK cell counts.

*In conclusion*, Zhi et al. provide valuable insights into the interplay between NK cells and CD20 + B-cell repopulation in SD/FRNS recurrence, highlighting the importance of immunological monitoring to optimize rituximab efficacy. However, the retrospective nature and heterogeneity of concomitant immunosuppressive regimens limit the study’s conclusions. Future prospective studies should evaluate the independent roles of NK cell dynamics, CD20 + B-cell kinetics, and immunosuppressive agents to clarify their contributions to disease recurrence and treatment outcomes.
